# Hepatitis A virus immunity and vaccination among at-risk persons receiving HIV medical care

**DOI:** 10.1016/j.pmedr.2018.06.006

**Published:** 2018-06-20

**Authors:** Nicholas P. DeGroote, Christine L. Mattson, Yunfeng Tie, John T. Brooks, Shikha Garg, John Weiser

**Affiliations:** a Oak Ridge for Science and Education, 1299 Bethel Valley Rd, Oak Ridge, TN 37830, United States; b Centers for Disease Control and Prevention, 1600 Clifton Rd, Atlanta, GA 30333, United States; c ICF International, 3 Corporate Blvd NE Suite 370, Atlanta, GA 30329, United States

**Keywords:** HIV, human immunodeficiency virus, HAV, hepatitis A virus, PLWH, persons living with HIV, MSM, men who have sex with men, PWID, persons who inject drugs, Human immunodeficiency virus, Hepatitis A, Vaccination, Men who have sex with men, Persons who inject drugs

## Abstract

United States guidelines recommend hepatitis A virus (HAV) vaccination for persons living with HIV (PLWH) who are at increased risk for HAV infection, including men who have sex with men (MSM) and persons who inject drugs (PWID). However, nationally representative estimates of vaccine coverage and immunity for this population are lacking. We used medical record and interview data from the 2009–2012 cycles of the Medical Monitoring Project, a nationally representative surveillance system of PLWH receiving HIV medical care in the United States, to estimate the prevalence of HAV immunity, defined as receipt of at least one dose of vaccine or laboratory documentation of anti-HAV antibodies, among 8695 MSM and PWID. Among HAV-nonimmune PLWH, we then examined factors associated with HAV vaccination during the 12-month retrospective observation period using Rao-Scott chi-square tests.

Among MSM and PWID receiving HIV medical care, 64% had evidence of HAV immunity. Among those who were nonimmune, 10% were vaccinated during the 12-month retrospective observation period. Factors associated with vaccination during follow-up included younger age (i.e., 18–29 years), self-reported black non-Hispanic race/ethnicity, having detectable HIV RNA, and having been diagnosed with HIV within the past 5 years. Over one third of MSM and PWID receiving HIV medical care during 2009–2012 cycles were not immune to HAV. This analysis suggests that a sizeable proportion of at risk MSM and PWID receiving HIV medical care do not receive HAV vaccination, which is currently recommended.

## 1 Introduction

Hepatitis A virus (HAV) infection is typically self-limited to acute liver inflammation and does not result in chronic illness (World Health Organization, 2015). However, HAV infection among persons living with HIV (PLWH) may lead to more severe illness and prolonged HAV viremia and shedding, which can lead to a longer infectious period (Ida et al., 2002). The most common non-AIDS related cause of death among PLWH in the United States is liver disease (Palella Jr. et al., 2006). An estimated 10% of PLWH in the United States are living with chronic hepatitis B virus infection and one-quarter with hepatitis C virus (HCV) infection, (Thio, 2009; Alter, 2006; Thio et al., 2002; Centers for Disease Control and Prevention, 2017) which can increase the risk of fulminant hepatitis and death from HAV infection (Vento et al., 1998). To reduce morbidity and mortality among PLWH with liver disease, HAV vaccination is a necessary and critical intervention. The Department of Health and Human Services' Guidelines for Prevention and Treatment of Opportunistic Infections in HIV-Infected Adults and Adolescents (Panel on Opportunistic Infections in HIV-Infected Adults and Adolescents, 2018) and the HIV Medicine Association's Primary Care Guidelines for the Management of Persons Infected with HIV (Aberg et al., 2014) recommend HAV vaccination for HIV-infected persons who inject drugs (PWID) and men who have sex with men (MSM).

Nationally representative estimates of HAV immunity and vaccine coverage for MSM and PWID living with HIV are lacking (World Health Organization, 2015; Centers for Disease Control and Prevention, 2016a). In this analysis we sought to estimate the prevalence of HAV vaccination, or anti-HAV antibodies conferring immunity, and to examine factors associated with HAV vaccination of non-immune PLWH for whom vaccination is recommended.

## 2 Methods

### 2.1 Sampling and data collection

We used data from the 2009–2012 cycles of the Medical Monitoring Project (MMP), a cross-sectional, complex sample survey designed to produce nationally representative estimates of behavioral and clinical outcomes among adults receiving outpatient HIV medical care in the United States and Puerto Rico (Centers for Disease Control and Prevention, 2016b). Further details on MMP's methods are described elsewhere (Centers for Disease Control and Prevention, 2016b). Briefly, during 2009–2012, MMP used a three-stage probability-proportional-to-size sampling method. First, states and territories were sampled, followed by outpatient facilities in those sampled states and territories providing HIV care, and finally, PLWH ages 18 years or older who had received medical care at participating facilities in those states and territories between January and April during the cycle year were sampled. All sampled states and territories participated. The facility response rates ranged from 76% to 85% and the patient response rates ranged from 49% to 55%. Data used in the analysis were collected from June 1, 2009 through May 31, 2013 via face-to-face or telephone interviews and medical record abstraction. Data were weighted for unequal selection probabilities and nonresponse. CDC determined the MMP to be a public health surveillance activity used for disease control, program, or policy purposes; however, some local institutional review board approval was obtained at participating states, territories, and facilities when required (Centers for Disease Control and Prevention, 2010). Informed consent was obtained from all interviewed participants.

### 2.2 Measures

HAV immunity was defined as having received at least one dose of an HAV vaccine or laboratory documentation of any positive detection of HAV antibodies (IgM, IgG, or total) in the patient's medical record. We defined patients not meeting these criteria as nonimmune and candidates to initiate HAV vaccination. Clinical characteristics included time since HIV diagnosis (categorized as <5 years vs ≥5 years); prescription of antiretroviral therapy (ART) in the past 12 months; and sustained viral suppression (all HIV RNA test result during the surveillance period undetectable or <200 copies/mL). Sociodemographic characteristics included being a PWID or an MSM; race/ethnicity (black/African American, non-Hispanic/Latino [black]; white non-Hispanic/Latino [white]; Hispanic/Latino; and other races/ethnicities); age; gender (male or female); educational attainment (<high school, high school or equivalent; or >high school); and whether the primary HIV care facility received funding from the Ryan White HIV/AIDS Program (RWHAP). The time reference for all measured variables were 12 months prior to the interview.

Among MSM and PWID without documentation of immunity at baseline, we estimated the percentage of patient vaccinated during the 12-month retrospective observation period. Twenty-seven MSM or PWID who had a positive HAV antibody result and lacked HAV vaccination documentation during the 12-month retrospective observation period were excluded from this analysis because they had developed natural immunity and were no longer candidates for vaccination. MSM and PWID were combined for this portion of the analysis due to the small number of PWID, many of whom were also MSM.

### 2.3 Data analyses

**Fig. 1:**
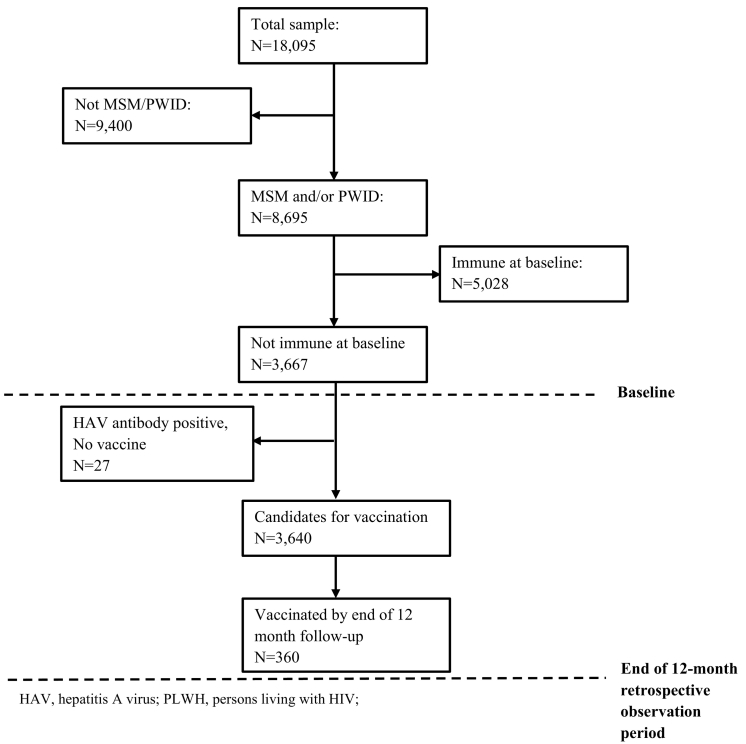
Participant distribution for factors associated with HAV vaccination of nonimmune persons receiving HIV care who are men who have sex with men (MSM) and/or persons who inject drugs (PWID), United States, 2009–2012.

The analytic dataset included records of 18,095 participants, of whom 8695 were MSM or PWID. We analyzed data across two time intervals: 1) from the date of HIV diagnosis until baseline at one year prior to the patient interview date; 2) from baseline through the patient interview date (a 12-month retrospective observation period) (Fig. 1). We computed frequencies, weighted percentages, and 95% confidence intervals (CI) to estimate the prevalence of HAV immunity at baseline among PLWH who were MSM, PWID, and MSM who were also PWID.

We computed frequencies and weighted percentages with their 95% confidence intervals (CI) to estimate the prevalence of HAV immunity at baseline among PLWH who were MSM, PWID, and MSM who are also PWID. We then estimated the overall prevalence of HAV immunity documented between the initiation of HIV medical care and baseline, and from baseline through the interview date. Finally, we assessed associations of vaccination during the 12-month retrospective observation period between baseline and the interview date among previously nonimmune patients with sociodemographic, behavioral, and clinical characteristics using the Rao-Scott chi-square test. Bivariate associations of patient characteristics with recent HAV vaccination were considered significant at *P* < 0.05. Analyses were completed using SAS 9.3 (SAS Institute, Cary, NC).

## 3 Results

Among persons receiving HIV medical care in the United States during June 2009 through May 2013, 48% were MSM, and 2% were PWID. Among MSM and PWID, the prevalence of HAV immunity at baseline was 58%. Specifically, the prevalence of HAV immunity among MSM was 57%, 60% among PWID, and 68% among MSM who were also PWID. Among MSM and PWID without documented immunity at baseline, 10% were vaccinated for HAV during the following 12 months. By the end of this observation period, 64% of MSM and PWID had documented immunity. Specifically, 64% of MSM had documented immunity, as did 65% of PWID, and 76% of MSM who were also PWID (Data not shown).

Table 1 displays sociodemographic and clinical characteristics of all US HIV patients and HIV patients who are MSM or PWID, stratified by hepatitis A immune status. Vaccination during the 12-month retrospective observation period was more common among younger persons, non-Hispanic blacks, and those born in the United States. Those more recently diagnosed with HIV, having a detectable HIV viral load test, and receiving care at a Ryan White HIV/AIDS Program funded facility were significantly more likely to be vaccinated during the 12-month retrospective observation period (Table 2).

**Table 1:** Prevalence of HAV immunity by sociodemographic characteristics for All HIV-infected patients, men who have sex with men or persons who inject drugs, medical monitoring project, United States, 2009–2012.

Characteristic	Overall (N = 18,095)				MSM or PWID (N = 8678)			
	Immune		Not immune		Immune		Not immune	
	n	% (95% CI)	n	% (95% CI)	n	% (95% CI)	n	% (95% CI)
Total	11,208	62 (59–65)	6887	38 (35–41)	5595	64 (61–68)	3083	36 (32–39)
Gender								
Male	8280	76 (73–78)	4780	70 (68–73)	5562	99 (99–100)	3063	99 (99–100)
Female	2762	24 (22–27)	2018	30 (27–32)	29	0.4 (0.2–0.6)	18	0.5 (0.3–0.8)
Sexual orientation								
Heterosexual or straight	5323	47 (42–51)	3601	51 (47–55)	146	2 (1–3)	71	2 (1–3)
Homosexual or gay	4748	43 (39–48)	2672	40 (36–44)	4604	83 (81–85)	2584	84 (82–86)
Bisexual	977	9 (8–9)	516	8 (7–8)	791	14 (12–16)	403	13 (12–15)
Other	160	2 (1–2)	98	1 (1–2)	54	1 (0.7–1.4)	25	1 (1–1)
Age								
18–29	857	8 (7–9)	486	7 (6–8)	530	10 (8–11)	249	8 (7–10)
30–39	1795	16 (15–17)	1046	15 (14–16)	947	17 (16–19)	473	15 (14–17)
40–49	3969	35 (34–36)	2427	35 (33–36)	2050	36 (35–38)	1116	36 (34–38)
≥50	4587	40 (39–42)	2928	43 (41–44)	2068	37 (35–39)	1245	40 (38–42)
Race/ethnicity								
White, non-Hispanic	3660	34 (29–40)	2234	35 (29–39)	2779	51 (45–57)	1585	53 (47–58)
Black, non-Hispanic	4677	42 (34548)	2799	41 (34–48)	1386	24 (19–29)	728	24 (20–29)
Hispanic or Latino	2307	19 (16–22)	1583	20 (14–26)	110	19 (16–21)	640	18 (14–23)
Other	564	5 (4–6)	271	4 (3–5)	343	6 (5–7)	134	5 (4–6)
Education								
<High school	2461	21 (19–23)	1515	21 (19–23)	524	9 (8–10)	266	9 (7–10)
High school diploma or equivalent	3063	27 (25–29)	1872	27 (25–29)	1226	22 (20−23)	639	21 (19–23)
>High school	5681	52 (49–55)	3496	52 (49–55)	3844	69 (67–71)	2177	71 (68–73)
Country or territory of birth								
Born in foreign country	1556	14 (12–16)	836	12 (11–14)	738	14 (12–15)	352	11 (9–13)
Born in US or PR	9647	86 (84–88)	6049	88 (86–89)	4855	86 (85–88)	2731	89 (87–91)
Homeless (past 12 months)								
Yes	1028	9 (8–10)	492	7 (6–8)	410	7 (6–8)	184	6 (5–7)
No	10,178	91 (91–92)	6394	93 (92–94)	5185	93 (92–94)	2899	94 (93–95)
Time since HIV diagnosis (yr.)								
<5 years	2383	22 (21–24)	1404	22 (20–23)	1261	24 (23–25)	626	21 (19–23)
≥5 years	8822	78 (76–79)	5476	78 (77–80)	4332	76 (75–78)	2454	79 (77–81)
Received STD screening								
Yes	7725	67 (63–70)	3620	50 (47–52)	3875	68 (65–71)	1646	52 (49–55)
No	3424	33 (30–37)	3193	50 (48–53)	1682	32 (29–35)	1397	48 (45–51)
Hepatitis C screening								
Yes	9652	85 (84–86)	4207	59 (56–61)	4753	85 (83–86)	1724	54 (52–57)
No	1556	15 (14–16)	2680	41 (39–44)	842	15 (14–17)	1359	46 (43–48)

HAV, hepatitis A virus; CI, confidence interval; MSM, men who have sex with men; PWID, persons who inject drugs; PR, Puerto Rico; STD, sexually transmitted disease.

**Table 2:** Factors associated with HAV vaccination of HAV nonimmune persons living with HIV who were men who have sex with men, persons who inject drugs, or both, 12-month retrospective observation period, United States, 2009–2012.

Characteristic	n^a^	% vaccinated (N = 3640)^b^	95% CI^b^	*P* value for Rao-Scott chi-square test
Total	360	10	8.1–11.9	

Age at time of interview (years)				
18–29	101	25.9	21.4–30.3	<0.0001
30–39	87	15.2	10.7–19.7	
40–49	103	8.4	6.0–10.8	
≥50	69	4.6	3.2–6.0	

Race/ethnicity				
White, non-Hispanic	155	8.4	6.2–10.6	<0.0001
Black, non-Hispanic	127	14.3	11.3–17.2	
Hispanic or Latino	59	8.1	5.4–10.7	
Other^c^	19	12	6.7–17.3	

Sexual behavior/orientation				
MSM	352	10	8.1–11.9	0.8
Non-MSM^d^	8	9.1	2.0–16.1	

Injection drug use				
Yes	19	12.1	6.2–18.0	0.43
No	341	9.9	8.1–11.8	

Education				
<High school	41	11.7	7.8–15.5	0.25
High school diploma or equivalent	89	11.2	8.3–14.1	
>High school	230	9.4	7.4–11.4	

Country or territory of birth				
Born in foreign country	23	4.7	2.6–6.8	0.0008
Born in United States or Puerto Rico	337	10.6	8.5–12.8	

Homeless at any time (during past 12 months)				
Yes	27	12.2	7.4–17.1	0.26
No	333	9.8	7.9–11.7	

Time since HIV diagnosis				
<5 years	211	23.0	19.1–26.9	<0.0001
5–9 years	42	6.8	4.2–9.3	
≥10 years	107	5.0	3.7–6.3	

Prescribed ART				
Yes	317	9.8	7.8–11.8	0.31
No	43	11.9	7.7–16.0	

Sustained viral suppression in the past 12 months				
Undetectable or <200 copies/mL	159	7.4	5.8–9.1	<0.0001
Detectable or ≥200 copies/mL	201	13.9	11.0–16.7	

Received care at a Ryan White HIV/AIDS Program funded facility				
Yes	251	14.3	12.3–16.4	<0.0001
No	64	4.1	2.5–5.6	

Abbreviations: HAV, hepatitis A virus; HIV, human immunodeficiency virus; CI, confidence interval; MSM, men who have sex with men; ART, antiretroviral therapy; STD, sexually transmitted disease.

a Frequencies are unweighted.

b Percentages and corresponding CIs are weighted percentages.

c Includes American Indian/Alaska Native, Asian, Native Hawaiian/Other Pacific Islander, or multiple races.

d Coefficient of variation >0.30 and this estimate may not be stable.

## 4 Discussion

In the general population, the incidence of HAV in the United States has dropped by 95% since the introduction of the HAV vaccine in 1995 (Centers for Disease Control and Prevention, 2016a). However, between 2000 and 2010, seroprevalence of HAV antibodies plateaued among persons aged 20–39 years and decreased among adults ≥40 (Murphy et al., 2016). Past studies have found that HAV antibody seroprevalence among PLWH ranged from 40 to 83% in MSM and from 62 to 86% in PWID (Lin et al., 2017). Although precise data are lacking, vaccine coverage among MSM and PWID in the general population appears to be low (Cotter et al., 2003).Our study was the first to examine HAV vaccination and immunity among people with increased risk for infection receiving HIV care at different types of facilities in both high and low prevalence areas, representative of the diversity of US HIV patients. In addition, our sampling and weighting methods permit inference to all US HIV patients at increased risk for HAV.

There are several plausible reasons for the low prevalence of HAV vaccination among patients receiving HIV medical care, including cost and lack of insurance reimbursement. The National Academy of Medicine reports substantial variation in vaccine coverage and payment policies among public and private insurers (Institute of Medicine Committee on Immunization Finance Policies and Practices, 2000). In addition, adults are not included in state systems for universal vaccine purchase and distribution. The RWHAP allows the use of funds to purchase and administer vaccines (Health Resources and Services Administration, 2017). However, the extent to which funds are used for this purpose is not known and at least one-quarter of HIV patients receive care at facilities that do not receive RWHAP funding (Weiser et al., 2015). Increasing vaccine coverage for PLWH could increase hepatitis A vaccination and reduce morbidity.

Providers might not vaccinate because they are unaware of vaccination guidelines or because they underestimate the risk and severity of HAV infection (Tenner et al., 2012; Nelson et al., 2017). The Advisory Committee on Immunization Practices has recommended hepatitis A vaccination of MSM and PWID since 1996 (Centers for Disease Control and Prevention, 1996). Since 1999, the US Public Health Service and the Infectious Disease Society of America have recommended vaccination all PLWH who are MSM or PWID (Centers for Disease Control and Prevention, 1999). Additional efforts to disseminate these guidelines are needed. Finally, concerns about vaccine safety, and patient unwillingness might be barriers to wider vaccination (Tenner et al., 2012). Clear messaging about the safety and effectiveness of hepatitis A vaccination is needed.

## 5 Study limitations

This study was subject to several limitations. First, because not all unvaccinated patients were tested for anti-HAV antibodies, our estimate of immunity may have been low. Second, because administration of HAV vaccine, particularly a single dose, is not always immunogenic, our estimate of the prevalence of immunity based on documented vaccination, may have been elevated. Nevertheless, more than a third of patients had a missed opportunity to at least begin the process of vaccination. Lastly, this analysis was limited to persons receiving HIV medical care, so our findings should not be generalized to all PLWH.

## 6 Conclusions

In conclusion, over a third of persons receiving HIV care in the United States had missed opportunities for HAV vaccination. Increased coverage for hepatitis A vaccination, wider dissemination of clinical guidelines, and clear messaging about the safety and effectiveness of the vaccine could boost vaccination rates among at-risk persons and prevent morbidity and mortality from HAV among PLWH.

## Funding source

This analysis was supported in part by an appointment to the Internship/Research Participation Program at the CDC, US Environmental Protection Agency, administered by the Oak Ridge Institute for Science and Education through an interagency agreement between the US Department of Energy and EPA. Funding for the Medical Monitoring Project is provided by the Centers for Disease Control and Prevention.

## Summary

A nationally representative estimation of hepatitis A virus (HAV) immunity and vaccination status suggests that a sizeable portion of at-risk persons receiving HIV medical care do not receive HAV vaccination per current guidelines.

## Disclaimer

The findings and conclusions in this report are those of the authors and do not necessarily represent the views of the Centers for Disease Control and Prevention (CDC).

## Conflict of interest

None.

## References

[bb0005] Aberg J.A., Gallant J.E., Ghanem K.G., Emmanuel P., Zingman B.S., Horberg M.A. (2014). Primary care guidelines for the management of persons infected with HIV: 2013 update by the HIV Medicine Association of the Infectious Diseases Society of America. Clin. Infect. Dis..

[bb0010] Alter M.J. (2006). Epidemiology of viral hepatitis and HIV co-infection. J. Hepatol..

[bb0015] Centers for Disease Control and Prevention (1996). Prevention of hepatitis A through active or passive immunization: recommendations of the Advisory Committee on Immunization Practices (ACIP). MMWR.

[bb0020] Centers for Disease Control and Prevention (1999). 1999 USPHS/IDSA guidelines for the prevention of opportunistic infections in persons infected with human immunodeficiency virus: U.S. Public Health Service (USPHS) and Infectious Diseases Society of America (IDSA). MMWR.

[bb0025] Centers for Disease Control and Prevention (2010). Distinguishing Public Health Research and Public Health Nonresearch. https://www.cdc.gov/od/science/integrity/docs/cdc-policy-distinguishing-public-health-research-nonresearch.pdf.

[bb0030] Centers for Disease Control and Prevention (2016). Hepatitis A Questions and Answers for Health Professionals. https://www.cdc.gov/hepatitis/HAV/HAVfaq.htm.

[bb0035] Centers for Disease Control and Prevention (2016). Behavioral and Clinical Characteristics of Persons Receiving Medical Care for HIV infection—Medical Monitoring Project, United States, 2014 Cycle (June 2014–May 2015). http://www.cdc.gov/hiv/library/reports/hiv-surveillance.html.

[bb0040] Centers for Disease Control and Prevention (2017). HIV and Viral Hepatitis. https://www.cdc.gov/hiv/pdf/library/factsheets/hiv-viral-hepatitis.pdf.

[bb0045] Cotter S.M., Sansom S., Long T. (2003). Outbreak of hepatitis A among men who have sex with men: implications for hepatitis A vaccination strategies. J. Infect. Dis..

[bb0050] Health Resources and Services Administration (2017). Ryan White HIV/AIDS Program Services: Eligible Individuals & Allowable Uses of Funds; Policy Clarification Notice #16-02. https://hab.hrsa.gov/program-grants-management/policy-notices-and-program-letters.

[bb0055] Ida S., Tachikawa N., Nakajima A. (2002). Influence of human immunodeficiency virus type 1 infection on acute hepatitis A virus infection. Clin. Infect. Dis..

[bb0060] Institute of Medicine Committee on Immunization Finance Policies and Practices (2000). Calling the Shots: Immunization Finance Policies and Practices. https://www.ncbi.nlm.nih.gov/books/NBK225581.

[bb0065] Lin K.Y., Chen G.J., Lee Y.L. (2017). Hepatitis A virus infection and hepatitis A vaccination in human immunodeficiency virus-positive patients: a review. World J. Gastroenterol..

[bb0070] Murphy T.V., Denniston M.M., Hill H.A. (2016). Progress toward eliminating hepatitis A disease in the United States. MMWR Suppl..

[bb0075] Nelson N.P., Allison M.A., Lindley M.C. (2017). Physician knowledge and attitudes about hepatitis A and current practices regarding hepatitis A vaccination delivery. Acad. Pediatr..

[bb0080] Palella F.J., Baker R.K., Moorman A.C. (2006). Mortality in the highly active antiretroviral therapy era: changing causes of death and disease in the HIV outpatient study. J. Acquir. Immune Defic. Syndr..

[bb0085] Panel on Opportunistic Infections in HIV-Infected Adults and Adolescents (2018). Guidelines for the Prevention and Treatment of Opportunistic Infections in HIV-infected Adults and Adolescents: Recommendations From the Centers for Disease Control and Prevention, the National Institutes of Health, and the HIV Medicine Association of the Infectious Diseases Society of America. http://aidsinfo.nih.gov/contentfiles/lvguidelines/adult_oi.pdf.

[bb0090] Tenner C.T., Herzog K., Chaudhari S., Bini E.J., Weinshel E.H. (2012). Knowledge, attitudes and barriers regarding vaccination against hepatitis A and B in patients with chronic hepatitis C virus infection: a survey of family medicine and internal medicine physicians in the United States. Int. J. Clin. Pract..

[bb0095] Thio C.L. (2009). Hepatitis B and human immunodeficiency virus coinfection. Hepatology.

[bb0100] Thio C.L., Seaberg E.C., Skolasky R. (2002). HIV-1, hepatitis B virus, and risk of liver-related mortality in the Multicenter Cohort Study (MACS). Lancet.

[bb0105] Vento S., Garofano T., Renzini C. (1998). Fulminant hepatitis associated with hepatitis A virus superinfection in patients with chronic hepatitis C. N. Engl. J. Med..

[bb0110] Weiser J., Beer L., Frazier E.L. (2015). Service delivery and patient outcomes in Ryan White HIV/AIDS Program-funded and -nonfunded health care facilities in the United States. JAMA Intern. Med..

[bb0115] World Health Organization (2015). Hepatitis A. http://www.who.int/mediacentre/factsheets/fs328/en.

